# Postoperative Pedicle Fracture in an Adult With Idiopathic Scoliosis After Posterior Spinal Fusion: A Case Report

**DOI:** 10.7759/cureus.87867

**Published:** 2025-07-13

**Authors:** Abdullah Alshebromi, Anas H Alshebromi, Mahdi Bassi

**Affiliations:** 1 Orthopedic Surgery, King Fahad Specialist Hospital, Qassim, SAU; 2 Orthopedic Surgery, Qassim Armed Forces Hospital, Qassim, SAU; 3 Orthopedics, Dr. Soliman Fakeeh Hospital, Jeddah, SAU

**Keywords:** adolescent idiopathic scoliosis, fracture, pedicle fracture, posterior spinal fusion, thoracolumbar orthosis

## Abstract

This case report presents the case of a 22-year-old female patient with a history of adolescent idiopathic scoliosis who underwent posterior spinal fusion and instrumentation from T4 to L5 at age 17. Her postoperative course was uneventful for five years, during which she remained asymptomatic and functionally independent. At age 22, she developed insidious axial low back pain without trauma or neurological symptoms. Imaging revealed a right L5 pedicle fracture with no evidence of implant loosening or pseudoarthrosis. Conservative management, including nonsteroidal anti-inflammatory drugs (NSAIDs), local injections, and physical therapy, failed to relieve symptoms. A CT scan confirmed solid fusion and an isolated pedicle fracture. Surgical removal of the instrumentation was performed, followed by application of a thoracolumbar orthosis. The patient returned to full daily activities without pain by one year postoperatively. This case highlights a rare complication of distal pedicle stress fracture after long-segment fusion, emphasizing the need for awareness of junctional stress-related pathology in long-term follow-up and the effectiveness of timely surgical management.

## Introduction

Scoliosis is a complex three-dimensional deformity of the spine, marked by a sideways curvature across multiple spinal segments and accompanied by vertebral rotation, leading to trunk misalignment and changes in the spine’s sagittal profile [1]. Based on its cause, scoliosis can be classified as idiopathic, congenital, or neuromuscular. The most prevalent type, adolescent idiopathic scoliosis (AIS), has no known origin, as indicated by the term "idiopathic" [2]. AIS is most often diagnosed in children or adolescents, particularly during periods of rapid growth. The global prevalence of AIS, defined by a Cobb angle greater than 10°, ranges from 0.93% to 12% [3]. It tends to occur more frequently in the female population, who also show a higher tendency for curve progression than males [4].

Management strategies for scoliosis include monitoring, bracing, and surgical intervention in more advanced cases. Treatment often requires surgical correction, particularly when the spinal curve is severe or worsening rapidly. If scoliosis is left untreated, it can lead to worsening spinal deformity over time. This progression may result in chronic back pain, lumbar nerve root compression (radiculopathy), visible physical deformities, neurological complications, and even limitations in heart and lung function. In particular, individuals with spinal curves exceeding 80° in the coronal plane may experience increased difficulty breathing [5]. In addition to these well-documented consequences, long-term mechanical complications such as pedicle fractures, though rare, may also occur following surgical correction, especially in long fusion constructs. One such complication is postoperative pedicle fracture, which can result from altered load distribution and increased biomechanical stress at the junctional zones of the fusion. These stresses are particularly pronounced in long constructs, where adjacent segments and posterior elements may be subjected to abnormal torque and strain, potentially leading to hardware failure, junctional kyphosis, or stress-related fractures [6]. Understanding these biomechanical implications is crucial for recognizing, preventing, and managing rare complications such as the one presented in this case report.

## Case presentation

**Figure 1:**
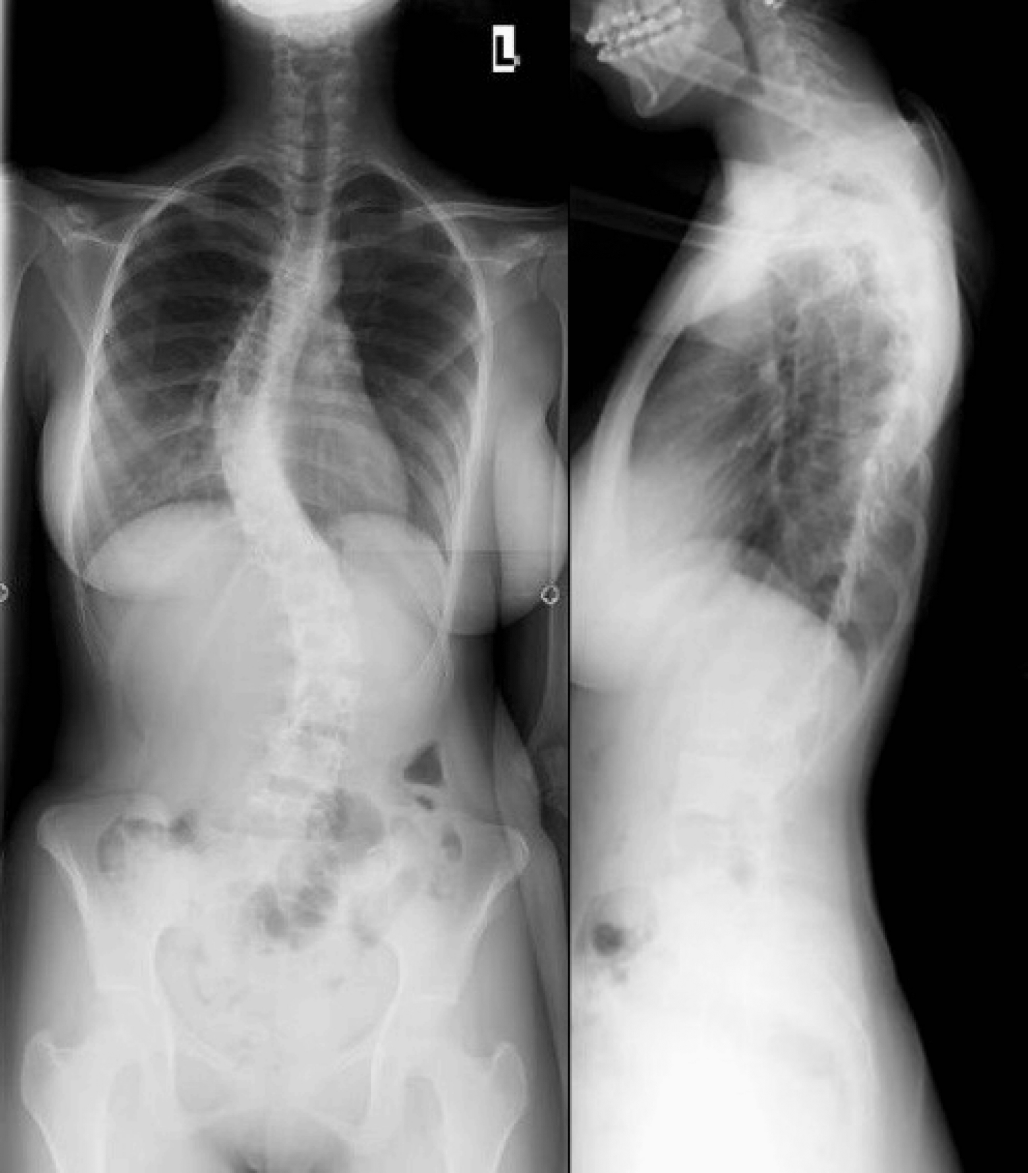
Preoperative anteroposterior and lateral chest and abdomen X-ray showing thoracolumbar kyphoscoliosis, characterized by a right thoracic curve measuring 61° and a left lumbar curve of 45°, forming an S-shaped deformity. There is no evidence of hemivertebrae or block vertebrae. A significant pelvic tilt of 60° is noted, and the Risser grade is 5, indicating skeletal maturity.

A 22-year-old female patient with a history of adolescent idiopathic scoliosis (AIS) presented to our spine clinic with complaints of gradual-onset axial low back pain. She had previously undergone posterior spinal fusion with instrumentation from T4 to L5 at the age of 17 due to progressive spinal deformity and gait imbalance. At the time of her initial presentation, accompanied by her parents, clinical examination revealed a flexible thoracolumbar scoliosis with a prominent right-sided rib hump. The Adam’s forward bending test accentuated the rib hump, and lateral bending confirmed curve flexibility. Standing whole-spine radiographs at the time demonstrated thoracolumbar kyphoscoliosis without congenital anomalies such as hemivertebrae or block vertebrae. The Cobb angle measured a 61° right thoracic curve and a 45° left lumbar compensatory curve. The pelvis was tilted at approximately 60°, and the Risser sign was Grade 5, indicating skeletal maturity (Figure 1). 

Following surgery, the patient was placed on a structured rehabilitation and follow-up protocol. Over the subsequent five years, she remained asymptomatic, engaged in routine activities of daily living, and reported no functional limitations. Radiographs during this period confirmed stable alignment and maintained instrumentation.

**Figure 2:**
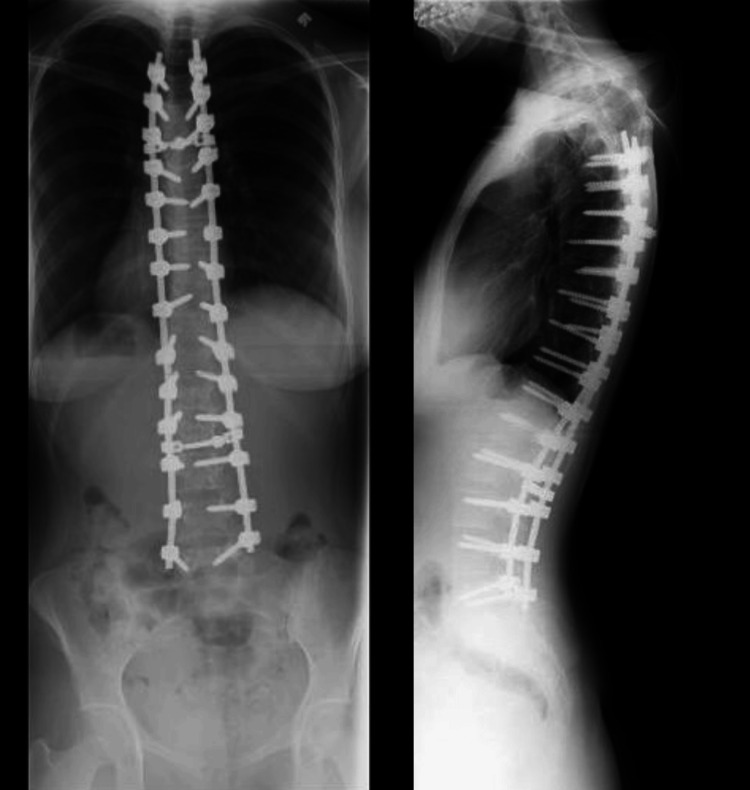
The postoperative radiographs, including posterior-anterior and lateral views, show well-aligned spinal instrumentation with pedicle screws and rods extending from the upper thoracic to the lower lumbar spine, indicating successful surgical correction of thoracolumbar scoliosis. The coronal and sagittal alignment appears well-maintained, and there is no radiographic evidence of hardware loosening, breakage, or migration. Spinal fusion appears intact, with stable fixation and preserved vertebral alignment, supporting the absence of mechanical complications despite the patient’s reported axial low back pain.

At age 22, she returned with complaints of dull, aching axial low back pain of six months’ duration. The pain was insidious in onset, localized to the lumbosacral area, rated 4-6/10 on the Visual Analog Scale (VAS), aggravated by prolonged sitting and physical exertion, and relieved partially by rest. There was no history of trauma, fever, weight loss, or night pain. She denied any radiculopathy, numbness, or weakness. On examination, she had localized tenderness over the lower lumbar spine without step-offs or gibbosity. Spinal range of motion was mildly restricted in flexion. Neurological assessment of the lower extremities, including motor strength, sensation, and reflexes, was normal. Repeat standing whole-spine radiographs showed no change in alignment and no evidence of hardware loosening, screw pull-out, or rod fracture (Figure 2).

**Figure 3:**
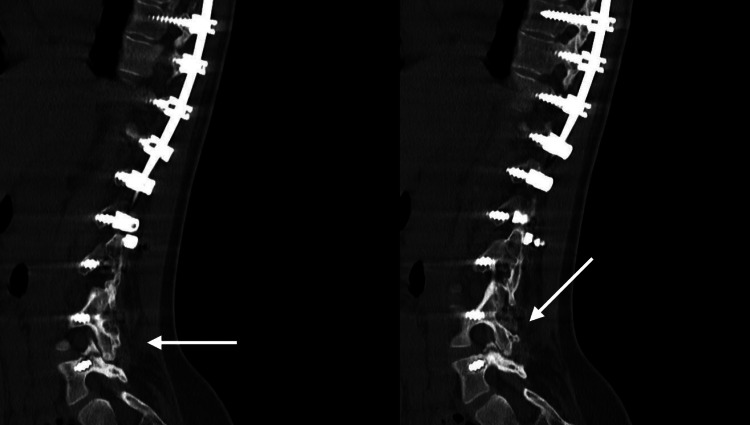
The CT scan revealed a well-fused instrumented spinal segment, confirming successful osseous fusion across the operated levels (arrows). However, it also identified a fracture of the right L5 pedicle, suggests a possible source of the patient’s axial low back pain, likely due to localized mechanical stress at the distal end of the instrumentation, despite the overall stability of the construct.

Conservative treatment, including oral analgesics, NSAIDs, local corticosteroid injections, and physiotherapy, was initiated but failed to provide relief. A subsequent CT scan revealed a well-fused instrumented segment but identified a fracture of the right L5 pedicle (Figure 3).

**Figure 4:**
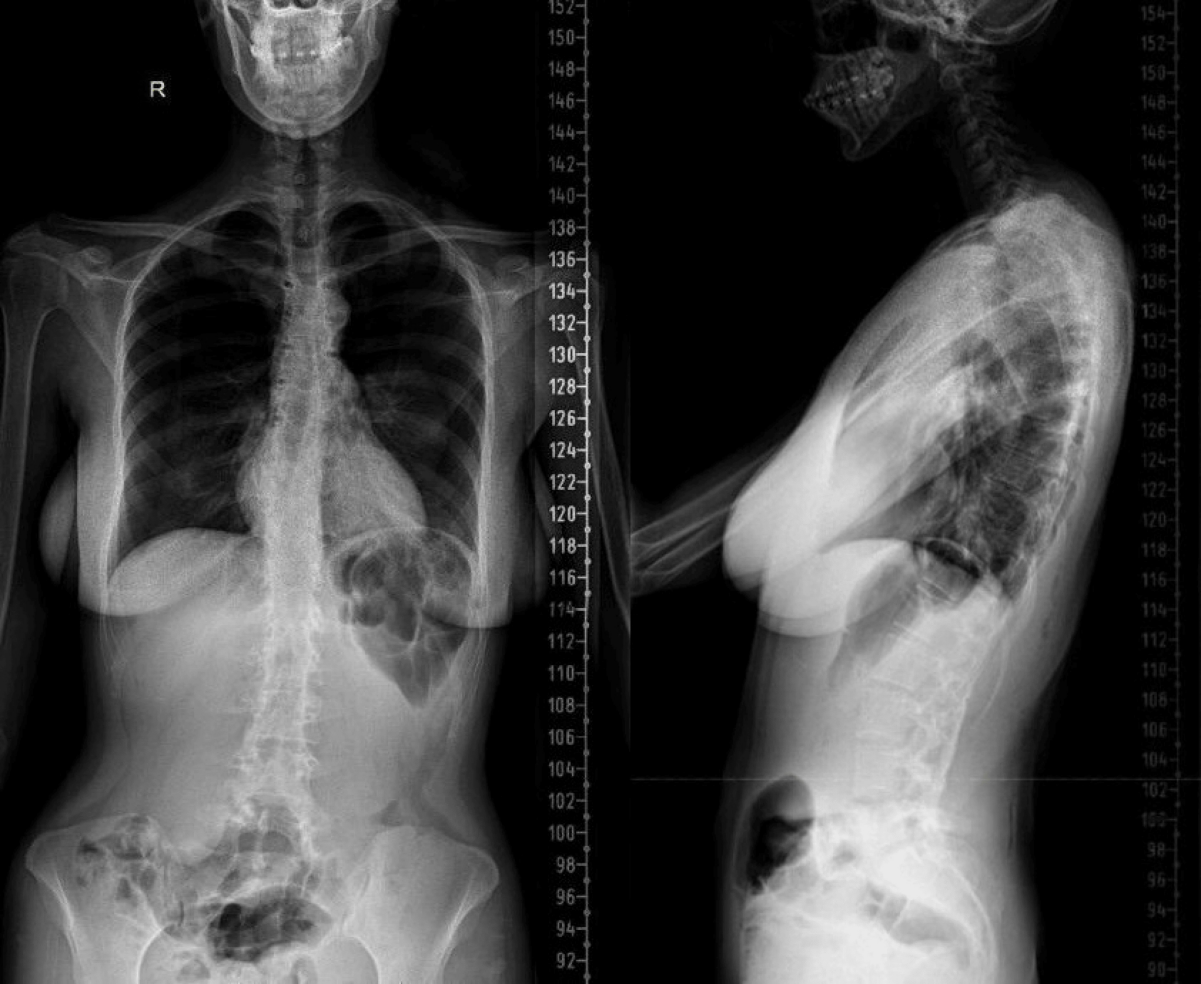
Anteroposterior and lateral radiograph shows the spine following removal of all implants, with no residual hardware visible. After surgical hardware removal and application of a thoracolumbar orthosis, the patient reported no symptoms at follow-up visits conducted at 6 and 12 weeks, and at 6 and 12 months.

As a result, she underwent surgical removal of all screws and rods, followed by the application of a thoracolumbar orthosis (Figure 4).

At follow-up visits at 6 and 12 weeks and 6 and 12 months post-surgery, she reported no symptoms and had resumed normal daily activities.

## Discussion

Adolescent idiopathic scoliosis (AIS) is the most common form of scoliosis, frequently requiring surgical intervention when curves progress beyond 40-50°. Posterior spinal fusion with segmental pedicle screw instrumentation remains a widely accepted treatment for severe curves, offering good deformity correction and long-term outcomes. In this case, a 22-year-old female had undergone posterior spinal fusion from T4 to L5 at age 17 for right thoracic (61°) and left lumbar (45°) scoliosis. The initial deformity was radiographically and clinically significant but correctable, with a Risser sign of 5 indicating skeletal maturity. 

In this case, the post-surgical course over five years was unremarkable, with no reported pain or neurological deficits, and she maintained her normal daily activities. However, at age 22, she developed insidious axial low back pain, and imaging revealed a right L5 pedicle fracture despite solid osseous fusion and no hardware failure. Pedicle fractures after long spinal constructs are rare and may reflect altered biomechanics at the caudal end of instrumentation, especially in long fusions that terminate at the lumbosacral junction. Comparatively, a study exploring surgical strategies for adult thoracolumbar scoliosis correction demonstrated that combined approaches such as transpsoas extreme lateral interbody fusion (XLIF) with posterior segmental instrumentation can offer superior radiographic correction (mean Cobb angle reduction from 38.5° to 10°) compared to posterior-only approaches. However, the combined techniques were associated with higher complication rates, including motor radiculopathy and sensory disturbances in up to 75% of patients [7]. In contrast, our patient's original posterior-only fusion yielded excellent long-term outcomes with minimal complications until the late-onset pedicle fracture. This highlights that even well-executed posterior spinal fusions are not without long-term biomechanical risks.

Although complications like proximal junctional kyphosis and adjacent segment degeneration are well recognized following long spinal fusions, pedicle fractures, particularly at distal levels, remain exceedingly rare. The literature indicates that pedicle stress fractures following lumbar fusion most commonly occur at the proximal or distal ends of the construct due to altered biomechanics and stress concentration at adjacent segments [8, 9]. In our case, the fracture at the right L5 pedicle occurred just distal to the end of the fusion, aligning with this known pattern.

Bilateral pedicle fractures have been previously documented in only a few cases, most of which were associated with prior spinal surgery or repetitive mechanical loading [8, 9]. Tribus and Bradford reported a similar case where a long posterior fusion from T3 to L4 led to a pedicle fracture at the lowest vertebral level (L4), emphasizing the vulnerability of terminal fusion levels to cantilever forces and junctional overload [9]. These biomechanical disturbances, induced by rigid constructs, can eventually lead to fatigue failure of posterior elements, even in the absence of trauma or implant failure.

In this case, the patient presented with no neurological deficits, and radiographs showed no implant loosening or breakage. Conservative treatment failed, and a CT scan confirmed the presence of a right L5 pedicle fracture with otherwise adequate fusion. The decision to remove the instrumentation and apply a thoracolumbar orthosis was based on symptomatology and structural integrity, which led to complete recovery over a year of follow-up.

Compared to typical postoperative outcomes in AIS patients, this case reflects a relatively favorable long-term result. Most individuals undergoing long-segment spinal fusion remain functionally independent and report good health-related quality of life, with minimal pain and disability in long-term follow-up studies [10]. Studies suggest that approximately 20-30% of AIS patients experience some degree of back pain years after surgery, nonetheless not always severe or functionally restrictive [5]. In this patient, the absence of radiographic or neurological abnormalities pointed toward mechanical causes of pain, such as paraspinal muscle fatigue or postural imbalance, rather than structural failure, implant-related complications, or adjacent segment disease.

Preventive strategies for late postoperative symptoms include continued physical rehabilitation focusing on core strength and flexibility, patient education to avoid high-impact activities, and regular long-term follow-up with imaging to detect biomechanical issues early. Thoughtful surgical planning, especially careful selection of the lowest instrumented vertebra, can help preserve motion segments and minimize stress at junctional zones, with potential benefits from limiting fusion short of the sacrum when appropriate.

## Conclusions

In conclusion, this case highlights a rare but important delayed complication: pedicle fracture, following long posterior spinal fusion for AIS. Despite this, the patient achieved a favorable outcome through timely diagnosis, conservative management with physical therapy, and close clinical monitoring. The success of the intervention underscores the value of individualized, non-operative strategies in cases lacking radiographic instability or neurological compromise. However, the case also has limitations, including the absence of quantitative outcome measures and lack of advanced biomechanical analysis to assess long-term stress distribution. Future studies should aim to incorporate objective functional assessments and biomechanical modeling to better understand risk factors and optimize long-term outcomes in post-fusion AIS patients.
